# Savouring Moderates Affect Reactivity to Daily Events in Old Age

**DOI:** 10.1002/smi.70030

**Published:** 2025-03-29

**Authors:** Shira Peleg, Miriam Wallimann, Theresa Pauly

**Affiliations:** ^1^ Department of Social and Health Sciences Bar‐Ilan University Ramat Gan Israel; ^2^ Department of Gerontology Simon Fraser University Vancouver Canada; ^3^ Department of Applied Social and Health Psychology University of Zurich Zurich Switzerland

**Keywords:** affect reactivity, daily diary, daily events, older adults, savouring

## Abstract

**Objective:**

This study investigated savouring as a moderator of affect reactivity to daily events among older adults.

**Method:**

A sample of 108 individuals aged 65–92 years (*M* = 73.11, *SD* = 5.92; 58% women) completed daily diary questionnaires over 14 days, reporting on daily stressors, positive events, savouring, and positive and negative affect.

**Results:**

Multilevel models showed that on days when a stressor was experienced, negative affect was higher when daily savouring (within‐person) was low, but this association was not observed when daily savouring was high. Additionally, on days with positive events, negative affect was lower when trait savouring (between‐person) was high, but this effect was not found when trait savouring was low.

**Discussion:**

The findings highlight the importance of savouring as a key factor in managing emotional responses to daily experiences among older adults. Specifically, deliberately engaging with positive experiences might buffer daily negative emotional responses.

## 1 Introduction

Affect reactivity, often conceptualised as the difference in affect (either positive or negative) on days when certain events are present compared to days when they are not (Almeida et al. 2023), plays a significant role in health outcomes. Reactivity to *daily stressors*, characterised by higher increases in negative affect and greater decreases positive affect in response to stressors, has been linked to both mental and physical health outcomes (e.g., Charles et al. 2013; Piazza et al. 2013; Sin et al. 2016, 2015), as well as to mortality (Chiang et al. 2018; Mroczek et al. 2015). Although less frequently researched, affect reactivity to *daily positive events*, characterised by higher increases in positive affect and greater decreases in negative affect in response to daily positive events, has also been linked to well‐being and emotional stability. In some studies, higher affect reactivity to positive events has been associated with negative outcomes including chronic pain, decreased well‐being, and lower emotional stability (e.g., Chen et al. 2022; Kircher et al. 2023; Grosse Rueschkamp et al. 2020). The fragility of positive affect hypothesis suggests that heightened reactivity to positive events may indicate greater event‐dependence of positive emotions and difficulty sustaining stable high levels of positive affect (Ong and Ram 2017). Yet, other research has linked heightened reactivity to positive outcomes. Specifically, stronger affective responses to positive experiences have been linked to higher well‐being (e.g., Carl et al. 2014), enhanced global physical and subjective well‐being (Lyubomirsky et al. 2005), and more pronounced positive affective reactions in non‐depressed individuals compared to those with depression (Bylsma et al. 2008).

These perspectives highlight the need for more research on situational and trait factors that moderate affective reactivity. This is especially important for older adults, who experience both adaptive and maladaptive changes in emotion regulation, cognitive functioning, and coping strategies (e.g., Charles 2010; Knight et al. 2007; Urry and Gross 2010), all of which can interact with affect reactivity in complex ways.

One factor that may be particularly relevant in old age is savouring, which refers to a conscious and deliberate process of engaging, focussing on, and appreciating everyday positive experiences in order to create, sustain, or amplify positive emotions (Bryant et al. 2011; Bryant and Veroff 2017). Socioemotional Selectivity Theory (SST; Carstensen et al. 1999) posits that as people age and perceive their time as limited, they prioritise the present and seek emotionally meaningful experiences. SST also suggests that older adults exhibit a positivity bias, marked by a tendency to focus on and remember more positive rather than negative stimuli (Carstensen et al. 1999). This bias aligns well with the core processes of savouring, which involve actively engaging with, concentrating on, and extending the enjoyment of positive experiences in the present moment.

## 2 Savouring and Affective Outcomes

According to Bryant and Veroff (2017), savouring involves both trait‐like and state‐like aspects. On one hand, individuals vary in their general tendency to savour positive experiences, which can be measured as a stable between‐person disposition. Simultaneously, savouring also functions as an emotion regulation strategy within specific daily situations, enhancing positive experiences as they occur.

Furthermore, the process of savouring can occur across three different time frames: one can savour by recalling past positive events, focussing on positive events in the presence, or anticipating and looking forward to upcoming positive events (Bryant and Veroff 2017). Regardless of the temporal dimension of the savouring strategy, the positive emotions felt during savouring are immediate and present (Bryant et al. 2011). In the present study, we examined both the personal (trait) and situational (state) aspects of savouring, with a particular emphasis on savouring in the present moment, which aligns with older adults' tendency to prioritise the present, driven by their limited future time perspective (Carstensen et al. 1999).

Although research on savouring among older adults is limited, recent work suggests that older adults are more likely to engage in savouring, and that this tendency is associated with greater positive emotions (Growney et al. 2024). Furthermore, it was found that savouring, in its trait form, was associated with older adults' subjective well‐being, including greater life satisfaction and happiness, and lower depression and unhappy mood (Bryant 2003; Smith and Hollinger‐Smith 2015). Moreover, although not investigated among older adults, a daily diary study has also found that daily savouring was positively associated with daily positive affect (Jose et al. 2012).

## 3 Savouring as a Moderator of the Effect of Daily Events on Affective Outcomes

Savouring may not only be associated with affective outcomes but can also serve as a moderator of affect reactivity to positive and negative events. Given its role as a regulator of the intensity and duration of positive affect in response to positive experiences (Bryant et al. 2011; Bryant and Veroff 2017), savouring is expected to amplify positive emotions in response to daily positive events. This amplification can occur because savouring involves actively attending to, enhancing, and prolonging the enjoyment of positive experiences, allowing individuals to extract greater emotional benefits from these moments (Bryant et al. 2011; Bryant and Veroff 2017).

Savouring may also play a role in stressor reactivity (Bryant and Veroff 2017). Specifically, individuals who frequently engage in savouring may cultivate a broader capacity to maintain positive affect even when faced with daily stressors. Savouring may also be conceptualised as a personal resource that enhances positive aspects of mixed‐emotion experiences or helps appraise ambiguous events as positive (e.g., a challenge; Sytine et al. 2019). This, in turn, may result in additional resource gains, such as positive affect and energy, for coping with negative events (Ma et al. 2020). For example, women with breast cancer showed higher relational well‐being on days on which they engaged in more capitalisation (retelling/sharing positive experiences with others; Otto et al. 2015). Therefore, it is expected that savouring would mitigate increases in negative affect in response to daily stressors and facilitate decreases in negative affect in response to daily positive events.

Some empirical support for these expectations exists. For example, it was found that individuals with a higher trait capacity for savouring reported higher positive and lower negative affect during positive events and higher positive affect during negative events (Ma et al. 2020). Other daily diary studies also found that daily savouring helped mitigate the negative effects of daily stressors on well‐being among adolescents (Sytine et al. 2019) and to moderate the effect of daily positive events on positive mood (Jose et al. 2012).

## 4 The Present Study

The present study investigated how savouring moderates the relationship between daily positive and negative events and their corresponding affective responses. In line with the concept that savouring encompasses both state and trait‐like elements, we investigate savouring as a resource that varies day‐to‐day and person‐to‐person. Moreover, the current study focused on older adults, recognising the potentially detrimental health impact of continuous exposure to stressors on this demographic group (Almeida 2005) and the relevance of savouring to their well‐being. While prior research has primarily focused on affect reactivity to stressors, and to a lesser extent on positive events, our study systematically investigates both. To achieve this purpose, we utilised an intensive‐longitudinal daily diary approach to capture daily experiences within the real‐life context of older adults.

Based on the abovementioned literature, it was hypothesised that: (1) experiencing daily stressors will be associated with lower positive affect and with higher negative affect on the same day, as compared to not experiencing any daily stressor; (2) higher daily and trait savouring will be associated with more beneficial affective reactivity to daily stressors (i.e., a weaker positive association between daily stressors and negative affect and a weaker negative association between daily stressors and positive affect); (3) experiencing daily positive events (uplifts) will be associated with higher positive affect and with lower negative affect on the same day, as compared to not experiencing any positive events; (4) higher daily and trait savouring will be associated with heightened affective reactivity to daily positive events (i.e., stronger negative association between daily positive events and negative affect and a stronger positive association between daily positive events and positive affect). The study was pre‐registered in January 2023 on the Open Science Framework. Hypotheses, rationale, and measures can be found at:

https://osf.io/kph6g.

Deidentified data and analysis code can be found at:

https://osf.io/93kts/.

## 5 Methods

### 5.1 Participants

Study participants included individuals aged 65 and above with a Swiss residency, who were enrolled in the ZWÄG 65+ study. This research project, an app‐based observational intensive‐longitudinal daily‐diary study, was conducted from June to September 2022. Recruitment methods involved both offline approaches, such as distributing study flyers, and online strategies, including reaching out through mailing lists. Out of the initial 123 individuals expressing interest, 108 participated and completed the study, representing an 88% participation rate. No participant withdrew from the study during the data collection phase. Participants' ages ranged from 65 to 92 (*M* = 73.11, SD = 5.93), with 58% of the sample identifying as women. All participants were retired. Regarding relationship status, 48% were married or in a committed relationship, 27% were divorced or in a dissolved partnership, 18% were widowed, and 7% were single. Forty‐eight percent reported living alone, while the remaining 52% reported living with a partner, other family members, or in assisted living. The majority of the sample (57%) graduated from lower secondary school, 40% held a higher‐secondary degree (Matura), and 3% held a primary/elementary school diploma. Self‐rated health was moderate‐to‐high (*M* = 3.74, SD = 0.79 on a 1–5 scale) in the present sample. Participants who completed the study were given the opportunity to participate in a lottery, offering prizes worth a total of CHF 500.

Based on an a‐priori‐power analysis, it was estimated that a sample of 92 participants was needed to ensure sufficient power (≥ 80%) for detecting moderate level‐1 (*r* = 0.3) and level‐2 (*r* = 0.3) effects. This calculation assumed an 85% adherence rate and moderate intra‐class correlation (Arend and Schäfer 2019). Our final sample, comprising *N* = 108 participants, yielded a power exceeding 99% for detecting moderate level 1‐effects and a power of at least 86% for detecting moderate level‐2 effects in the current study.

### 5.2 Procedure

Data was collected as part of the study ZWÄG 65+ (Wallimann et al. 2024), which was approved by the Ethics Committee of the University of Zurich. After a telephone screening, eligible participants were invited to a group baseline appointment (consisting of 1–5 participants) at the University of Zurich. During this session, participants provided informed consent, and the ‘Ethica’ app (Avicenna Research, version 5.02; 2022) was installed on their personal mobile devices, tablets, or provided devices. The baseline questionnaire was administered (and included sociodemographic, health‐related, and personality‐related information), and participants received instructions for the upcoming daily diary period, which started the following day. The third part of the study involved the daily diary period: Over 14 consecutive days, participants were asked to complete an end‐of‐day diary before bedtime, addressing questions related to affect, daily stressors, savouring, and other variables not covered in the present study, such as time spent in solitude and physical symptoms (the full list of measures can be found on OSF; https://osf.io/93kts/). Automatic reminders were sent at 8:30 p.m. and 9 p.m. The final part of the study included a questionnaire that was sent through the app the day after the daily diary completion. This questionnaire served as a debriefing and to collect participants' feedback on study participation. On average, participants reported that the mobile device was relatively easy to use (*M* = 9.44, SD = 0.94 on a 10‐point scale) and perceived the study participation period as typical for their daily lives (*M* = 7.46, SD = 2.72 on a 10‐point scale). High compliance with end‐of‐day diaries was observed, with participants answering an average of 13.23 (SD = 1.16, range: 9–14) out of 14 end‐of‐day diaries.

### 5.3 Measures

#### 5.3.1 Background Variables

Participants provided information on their age, gender (0 = men; 1 = women), living situation (0 = living with others; 1 = living alone), and highest educational attainment (0 = primary or secondary education; 1 = higher‐secondary education). In addition, participants' self‐rated health (SRH) was measured by indicating one's health status in general, on a scale ranging from 1 (poor) to 5 (excellent).

#### 5.3.2 Daily Events

Daily events included both measures of daily stressors and daily positive events (uplifts). *Daily stressors* were measured by a modified version (Klaiber et al. 2021) of the Daily Inventory of Stressful Events (Almeida et al. 2002). Participants were requested to specify the domain in which they encountered a stressful event on the given day. The listed domains included: ‘Argument, conflict, or disagreement’; ‘Family/home‐related’; ‘Financial problems’; ‘Traffic or transportation’; ‘Health problem or accident’; ‘Stressful event that happened to close friends or family’; and ‘Other stressful event’. *Daily positive events (uplifts)* were measured by asking participants to specify the domain in which they experienced a positive event on a given day. The listed positive events included: ‘Positive social interaction, in‐person’; ‘Positive social interaction, remote’; ‘Positive event at work, school, or volunteer position’; ‘Positive event at home’; ‘Positive event that happened to a close friend or family member’; ‘Spent time enjoying or viewing nature’; ‘Other positive event’ (Klaiber et al. 2021; Sin and Almeida 2018).

Based on previous work on affect reactivity to daily events (e.g., Schilling et al. 2022; Sin et al. 2015, 2020) and due to the low and skewed number of stressors and the high number of positive events older adults typically report (Zautra et al. 2005), dummy variables were calculated for daily stressors and daily positive events, in which 0 = no stressor/no positive event was experienced during that day, and 1 = at least one stressor/one positive event was experienced during that day.

#### 5.3.3 Affect

Positive and negative affect was measured based on the circumplex model of affect (Mak and Schneider 2022; Russell 1980). Participants were presented with the statement ‘Since waking up today, I felt…’ along with a set of 12 affect items. Six of the items represented positive affect (‘happy’, ‘excited’, ‘enthusiastic’, ‘calm’, ‘relaxed’, and ‘content’) and the other six items represented negative affect (‘distressed’, ‘frustrated’, ‘tense’, ‘unhappy’, ‘sad’, and ‘depressed’). Participants were then instructed to rate the extent they felt this way for each item on a scale ranging from 0 (not at all) to 100 (very much). Scores were computed by averaging the relevant items, with higher scores representing higher positive respectively negative affect. Reliabilities were assessed using reliability scores for intensive longitudinal data. RkF (Reliability over k Fixed days) measures the reliability of affect by averaging scores across the 14 research days to reflect person‐level differences, and RC (Reliability of Change) evaluates the measure's ability to detect systematic day‐level changes over that period. Both scales showed high reliability within and between persons (RkF = 0.99, Rc = 0.78 for positive affect, and RkF = 0.99, Rc = 0.80 for negative affect).

#### 5.3.4 Savouring

Daily savouring was measured by three items of the Savour‐4 questionnaire (Bryant et al. 2021): ‘I consciously acknowledged my positive feelings to myself’, ‘I deliberately focused my attention on my positive feelings’, and ‘I thought or did something that increased my positive feelings’. These items were assessed in relation to participants' reported daily positive events (see the ‘Daily events’ measures).^1^ Specifically, participants were asked to indicate the extent to which each of these items applied to today's positive experiences, on a scale ranging from 0 (not at all) to 100 (very much). Scores were recoded to 0–10 and a total score for daily savouring was computed by averaging the three items (*M* = 5.07, SD = 2.76; RkF = 0.99, Rc = 0.77). *Trait savouring* was calculated by averaging daily savouring scores for each participant across the measurement days. This method for calculating trait savouring is based on studies suggesting that the concept of stable individual characteristics can be seen as an individual's distributions of states (e.g., Fleeson 2001; Ram and Gerstorf 2009; Willroth et al. 2020). Furthermore, there is evidence that averaged daily measures are as strong, or more strongly associated with relevant health and well‐being outcomes (Ferguson et al. 2024; van Geusau et al. 2021).

### 5.4 Statistical Analyses

All analyses were conducted using the R (4.3.0) software (R Core Team 2023). To account for the hierarchical structure of the data (assessment days nested within individuals), multi‐level models were applied with assessment days as level 1 and individuals as level 2. Four models were examined. In the first two models—main effects only—we included dummy‐coded daily positive events and daily stressors (0 = no positive/negative event was experienced, 1 = at least one positive/negative event was experienced) and within‐person‐centred daily savouring at the within‐person level and person‐average means of daily variables at the between‐person level (i.e., the proportion of days out of the 14‐day study period an individual reported any positive events/stressors and average savouring across the 14‐day study period, centred on the sample mean) to predict positive and negative affect. In a second set of models, four interaction effects were entered: (1) daily positive events and daily savouring (within‐person; ‘Daily positive events × Daily savouring’); (2) daily stressors and daily savouring (within‐person; ‘Daily stressors × Daily savouring’); (3) daily positive events and trait savouring (cross‐level interaction; ‘Daily positive events × Trait savouring’); and (4) daily stressors and trait savouring (cross‐level interaction; ‘Daily stressors × Trait savouring’). All models also included control variables (age, gender, education, living situation, and self‐rated health). However, because the pattern of results remained the same with and without control variables, results are reported for models without control variables. Model equations were as follows.

Level 1:

$$\begin{array}{ll} {\text{Affect}}_{it}= & \beta_{0i}+\beta_{1i}\;\left({\text{Daily}\;\text{Positive}\;\text{Event}}_{it}\;\right)\\ & +\beta_{2i}\;\left({\text{Daily}\;\text{Stressor}}_{it}\right)\\ & +\beta_{3i}\;\left({\text{Daily}\;\text{Savouring}}_{it}\right)\\ & +\beta_{4i}\;\left(\text{Daily}\;\text{Positive}\;\text{Event}\times {\text{Daily}\;\text{Savouring}}_{it}\right)\\ & +\beta_{5i}\;\left(\text{Daily}\;\text{Stressor}\times D{\text{aily}\;\text{Savouring}}_{it}\right)\\ & +\beta_{6i}\;\left({\text{Study}\;\text{Day}}_{it}\right)+e_{it} \end{array}$$

where *i* represents the individual, *t* represents the day. $\beta_{1i}$ and $\beta_{2i}$ capture affect reactivity to stressors and positive events, respectively. $\beta_{3i}$ represents the effect of daily savouring, $\beta_{6i}$ accounts for time trends across study days, and $e_{it}$ is the residual error.

Level 2:

$$\beta_{0i}=\gamma_{00}+\gamma_{01}\;\left(\text{Trait}\;{\text{Savouring}}_{i}\right)+\gamma_{02}\;\left(\text{Average}\;\text{Stressor}\;{\text{Exposure}}_{i}\right)+\gamma_{03}+\left({\text{Average}\;\text{Positive}\;\text{Event}\;\text{Exposure}}_{i}\right)+u_{0i}$$

where $\gamma_{01}$ represents the effect of trait savouring on the intercept, $\gamma_{02}$ and $\gamma_{03}$ represent effects of average stressor and positive event exposure on the intercept, respectively, and $u_{0i}$ is the random intercept.

$$\beta_{1i}=\gamma_{10}+\gamma_{11}\;\left(\text{Trait}\;{\text{Savo}u\text{ring}}_{i}\right)+u_{1i}$$

$$\beta_{2i}=\gamma_{20}+\gamma_{21}\;\left(\text{Trait}\;{\text{Savo}u\text{ring}}_{i}\right)+u_{2i}$$

$$\beta_{3i}=\gamma_{30}+u_{3i}$$

$$\beta_{4i}=\gamma_{40}$$

$$\beta_{5i}=\gamma_{50}$$

$$\beta_{6i}=\gamma_{60}$$

## 6 Results

Overall, participants completed 1429 daily diaries. Thirty‐seven percent of the variance in both positive and negative affect was attributable to within‐person variability. Table 1 presents the study variables' means, standard deviations, and the between‐ and within‐person correlations. As can be seen, at least one stressor was experienced on 32% of the days, and at least one positive event was experienced on 95% of the days. About 2/3rd of the variance in positive and negative affect was due to differences between persons (${\text{ICC}}_{\text{positiveaffect}}$ = 0.63, ${\text{ICC}}_{\text{negativeaffect}}$ = 0.67), whereas about 1/3rd of the variance could be attributed to meaningful within‐person affect fluctuations and level‐1 measurement error. Higher savouring was associated with higher positive affect, both at the between and the within‐person level, and experiencing at least one stressor was associated with lower positive affect, both at the between and the within‐person level. Likewise, experiencing at least one stressor, but not savouring, was associated with a higher negative affect (savouring was associated with a higher negative affect only at the within‐person level, and this correlation was small). Positive events were associated with neither positive nor negative affect at the between‐person level, and at the within‐person level, the associations were small.

**TABLE 1:** Means, standard deviations, and between‐person (within‐person) correlations among the study variables (*N* = 108 participants).

		*M* (SD)/%	Age	Gender	Education	Living situation	SRH	1	2	3	4	5
1	Positive affect	62.94 (19.45)	−0.05	−0.15	0.08	−0.20*	0.33**	1				
2	Negative affect	12.60 (16.19)	0.01	0.16	−0.04	0.18	−0.19*	−0.50*** (−0.57***)	1			
3	Daily positive events^a^ (1 = at least one positive event was experienced during that day)	95%	−0.12	−0.04	0.25	0.15	0.12	0.13 (0.12***)	−0.05 (−0.14***)	1		
4	Daily stressors^a^ (1 = at least one stressor was experienced during that day)	32%	0.04	0.11	0.01	0.07	−0.36*	−0.42*** (−0.24***)	0.51*** (0.30***)	0.05 (−0.01)	1	
5	Daily savouring	5.07 (2.76)	0.05	0.24	0.14	0.19	0.17	0.49*** (0.41***)	0.07 (−0.23***)	0.17 (0.11***)	−0.04 (−0.10***)	1

*Note:* Age was measured in years. Gender was coded as 0 = Men, 1 = Women. Education, based on highest educational attainment, was coded as 0 = primary or secondary education, 1 = higher‐secondary education. Living situation was coded as 0 = Living with others, 1 = Living alone. Self‐rated health (SRH) was measured on a scale ranging from 1 (poor) to 5 (excellent).

^a^ The percentage of ‘Daily stressors’ and ‘Daily positive events’ was calculated as the number of days on which participants indicated experiencing at least one stressor/positives event respective to all measurements obtained.

^*^ *p* < 0.05.

^**^ *p* < 0.01.

^***^ *p* < 0.001.

### 6.1 Daily Events and Savouring as Predictors of Positive and Negative Affect

Table 2 presents the results of multilevel analyses for predicting positive and negative affect by daily events (daily stressors and positive events) and savouring. On days when participants engaged in more daily savouring or experienced daily positive events, they reported *higher* positive affect. In addition, experiencing daily stressors was associated with *lower* positive affect on the same day. At the between‐person level, higher trait savouring was associated with higher average positive affect and greater overall stress‐exposure was associated with decreased average positive affect. Similarly, experiencing daily stressors was associated with *higher* negative affect, while engaging in more daily savouring was associated with *lower* negative affect on the same day. Overall higher stressor exposure was associated with increased average negative affect.

**TABLE 2:** Fixed and random effects estimates for multilevel models predicting affective outcomes by daily events and savouring (*N* = 108).

Variable	Positive affect	Negative affect
	*b* (SE)	*b* (SE)
Fixed effects		
Intercept	54.89*** (11.78)	12.45 (10.17)
Study day	0.02 (0.07)	−0.02 (0.06)
Daily positive events	5.82** (1.93)	−3.77 (1.95)
Person‐average daily positive events	10.59 (11.69)	−4.00 (10.43)
Daily stressors	−5.23*** (0.89)	5.98*** (0.75)
Person‐average daily stressors	−19.25*** (4.54)	17.92*** (4.09)
Daily savouring	2.68*** (0.24)	−1.21*** (0.24)
Trait savouring (person‐average savouring)	3.90*** (0.53)	−0.25 (0.47)
Random effects (SD)		
Intercept	16.75 [12.55; 18.65]	*12.93* [9.43; 16.85]
Daily stressors	5.42 [3.23; 7.42]	4.59 [2.93; 6.18]
Daily positive events	6.60 [2.87; 11.44]	8.62 [4.06; 13.42]
Daily savouring	1.64 [0.09; 2.14]	1.85 [1.40; 2.33]
Residual	9.83 [9.43; 10.25]	8.24 [7.90; 8.59]
Explained variance		
Marginal *R* ^2^/conditional *R* ^2^	0.36/0.76	0.19/0.73

*Note:* Marginal *R* ^2^ denotes the variance explained by fixed effects, and conditional *R* ^2^ denotes the variance explained by both fixed and random effects in the model. We used profile likelihood based estimation for the confidence intervals for the random effects with the confint function in *R*.

*p < 0.05.

^**^ *p* < 0.01.

^***^ *p* < 0.001.

**FIGURE 1:**
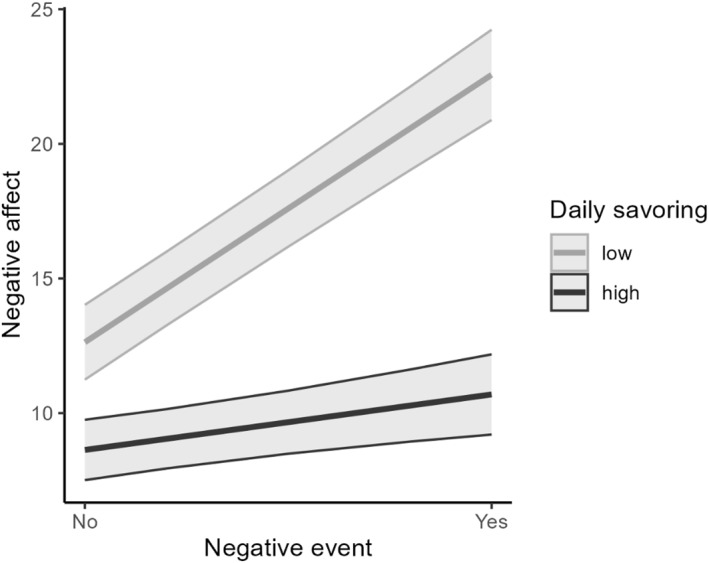
Illustration of the daily stressors × daily savouring interaction on negative affect.

**FIGURE 2:**
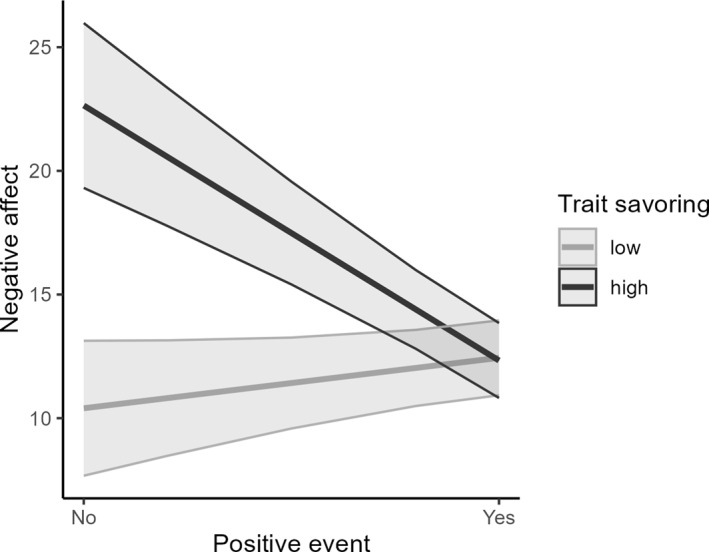
Illustration of the positive daily events × trait savouring interaction on negative affect.

Table 3 presents the results of the multilevel analysis with the interactions between daily events and savouring (both as in its state and trait form). Negative affect reactivity to daily stressors was moderated by daily savouring (Figure 1). Simple slopes analyses indicated that experiencing a daily stressor was associated with higher same‐day negative affect when levels of daily savouring were low (*b* = 9.93, SE = 1.20, *p* < 0.001), but not when levels of daily savouring were high (*b* = 2.06, SE = 1.21, *p* = 0.09). Furthermore, a significant interaction was found with respect to trait savouring moderating negative affect reactivity to positive daily events (Figure 2). Specifically, simple slopes analysis indicated that experiencing a daily positive event was associated with lower same‐day negative affect when levels of overall savouring were high (*b* = −10.31, SE = 3.08, *p* = 0.002), but not when levels of overall savouring were low (*b* = 2.03, SE = 2.44, *p* = 0.41). No significant interaction between daily events and daily savouring was found for positive affect as the outcome.

**TABLE 3:** Fixed and random effects estimates for multilevel models predicting affective outcomes by daily events, savouring, and their interactions (*N* = 108).

Variable	Positive affect	Negative affect
	*b* (SE)	*b* (SE)
Fixed effects		
Intercept	54.14*** (11.80)	13.31 (10.11)
Study day	0.02 (0.07)	−0.01 (0.06)
Daily positive events	6.21** (1.98)	−4.13 (2.11)
Person‐average daily positive events	11.21 (11.81)	−5.23 (10.50)
Daily stressors	−5.20*** (0.90)	5.99*** (0.76)
Person‐average daily stressors	−20.01*** (4.59)	19.74*** (4.11)
Daily savouring	2.88*** (0.78)	−1.26 (0.72)
Trait savouring (person‐average savouring)	2.74* (1.08)	2.68** (0.92)
Daily positive events × daily savouring	−0.37 (0.78)	0.56 (0.70)
Daily stressors × daily savouring	0.41 (0.40)	−1.42*** (0.34)
Daily positive events × trait savouring	1.14 (0.89)	−2.83** (0.83)
Daily stressors × trait savouring	−0.38 (0.42)	0.37 (0.35)
Random effects (SD)		
Intercept	15.80 [11.16; 17.66]	12.44 [8.82; 16.60]
Daily stressors	5.44 [3.17; 7.37]	4.74 [3.12; 6.24]
Daily positive events	5.80 [0.18; 10.54]	10.35 [4.62; 11.58]
Daily savouring	1.62 [0.00; 2.12]	1.85 [1.41; 2.31]
Residual	9.84 [9.43; 10.25]	8.17 [7.84; 8.51]
Explained variance		
Marginal *R* ^2^/Conditional *R* ^2^	0.36/0.76	0.22/0.74

*Note:* Marginal *R* ^2^ denotes the variance explained by fixed effects, and conditional *R* ^2^ denotes the variance explained by both fixed and random effects in the model. We used profile likelihood based estimation for the confidence intervals for the random effects with the confint function in *R*.

^*^ *p* < 0.05.

^**^ *p* < 0.01.

^***^ *p* < 0.001.

## 7 Discussion

The aim of the present study was to investigate the potential role of savouring for modulating affective experiences associated with positive and negative daily events using an intensive‐longitudinal daily diary approach. Overall, savouring in the present study was found as a valuable resource in older age for everyday well‐being. Specifically, beyond its association with overall higher positive affect, daily savouring was found to buffer the positive association between experiencing daily stressors and increased negative affect and higher trait savouring was found to amplify the negative association between experiencing daily positive events and decreased negative affect.

### 7.1 Affect Reactivity to Daily Events Among Older Adults

Consistent with a large body of research (e.g., Bolger et al. 1989; Mroczek and Almeida 2004; Schönfeld et al. 2016; Smyth et al. 1998), the findings in the present study, obtained both from the within and between person correlations and predictive models, indicated that daily stressors are associated with both higher same‐day negative affect and lower same‐day positive affect. The findings regarding daily positive events, however, are less straightforward. Specifically, in the present study, daily positive events were found to be associated with higher positive and lower negative affect, but only at the within‐person level and with small correlations. The null effect on the between‐person level contradicts other work that found both within‐ and between‐person effects of daily positive event frequency on positive affect (e.g., Charles et al. 2016; Chen et al. 2022; Zautra et al. 2005). However, these studies included participants with a wider age range and only a small proportion of older adults. Indeed, Röcke et al. (2009) found that older adults are less reactive to daily positive events than young adults and suggested that older adults' tendency to report higher positive affect and life satisfaction may be related to their lower affect reactivity, resulting in more balanced and stable daily experiences.

However, participants in our sample *did* show strong affective correlates of daily stressors. One reason for a more pronounced reaction to stressors (and not positive events) may be found in event intensity (Charles 2010). That is, the intensity of the stressors may have been perceived as stronger than the intensity of the positive events experienced, and thus provoked a stronger affective response. Indeed, it was found that event intensity explained an average of 14.8% of the total variance in momentary affect (Wenzel et al. 2022). Future studies are thus advised to consider event intensity in order to examine this explanation.

### 7.2 Savouring, Daily Events, and Affective Outcomes

Consistent with previous studies (e.g., Bryant 2003; Jose et al. 2012; Smith and Hollinger‐Smith 2015), findings indicated that savouring was associated with higher same‐day positive affect and with lower same‐day negative affect. Furthermore, consistent with the concept of savouring as a strategy for up‐regulating positive emotions, findings of the present study indicate that its association with positive affect is stronger than its association with negative affect (at the between‐ and within‐person level).

Beyond the association between savouring and affect, the findings of the present study also highlight the moderating role of savouring on affect reactivity. Specifically, daily savouring buffered the association between experiencing stressors and increased negative affect and amplified the association between experiencing positive events and decreased negative affect. These findings are consistent with previous research indicating that savouring acts as a moderator for affect reactivity (Jose et al. 2012; Ma et al. 2020; Sytine et al. 2019).

Empirical research remains unclear as to whether heightened positive affective reactivity is adaptive or reflects an inability to sustain high levels of positive affect, potentially indicating positive affect fragility (Ong and Ram 2017). In line with this, other positive psychological resources, such as a sense of purpose, have been found to be associated with a smaller increase in positive affect on days with positive events, compared to days without such events (Hill et al. 2022).

The finding that savouring moderated negative affect reactivity suggests that it could help mitigate stress or negative emotions. Unlike traditional coping strategies that focus on managing or reducing the impact of stressors (Folkman et al. 1986; Monat and Lazarus 1991), savouring aims to enhance positive experiences and emotions (Bryant et al. 2011; Bryant and Veroff 2017). This distinguishes savouring from coping mechanisms like reappraisal. While savouring and cognitive reappraisal both aim at reducing negative affect, they operate differently. Cognitive reappraisal modifies the interpretation of an event to reduce its emotional impact, often by reframing a stressor in a less threatening way (van 't Riet and Ruiter 2013). In contrast, savouring focuses on amplifying and prolonging positive emotional experiences rather than altering the appraisal of the event itself (Bryant and Veroff 2017). Thus, while savouring may provide an emotional buffer against stress, it does so by enhancing existing positive emotions rather than by changing the perceived meaning of a negative event. Furthermore, although savouring is also distinct from avoidance strategies that seek to escape or ignore stressors (van 't Riet and Ruiter 2013), it may function as an active distraction strategy, which has been shown to be effective in regulating negative emotions (Webb et al. 2012). Further research is needed to explore this potential similarity, particularly by investigating whether savouring occurs in response to or independently of stressors.

From a practical standpoint, these findings underscore the potential of savouring as a valuable tool for mitigating negative affect associated with daily events among older adults. Existing savouring‐based interventions conducted among older adults have demonstrated positive outcomes, such as improvements in depressive symptoms, life satisfaction, and happiness levels (e.g., Ho et al. 2014; Smith and Hanni 2017). Our findings further suggest that savouring daily positive experiences could be an important resource for future interventional research directed at decreasing negative affectivity among older adults.

It is important to note, however, that the moderating effects of savouring in the present study were observed only for affect reactivity to daily events in terms of negative affect but not positive affect. Thus, while savouring on a general level is more strongly related to levels of positive affect (as can be seen by its stronger associations with positive affect rather than negative affect), its ability to *modulate* affective fluctuations in older adults in responses to daily stressors and positive experiences could apply only in the case of negative but not positive affect. Older adults report a preference for high stable levels of positive affect (Charles 2010; Palmer and Gentzler 2019; Riediger et al. 2009; Smith and Hanni 2019). Indeed, studies have revealed that advancing age is associated with an inclination to maintain positive affect when individuals already express positive feelings. However, it is negatively correlated with the inclination to intensify positive affect (Riediger et al. 2009). Smith and Hanni (2019) found that interventions aimed at increasing savouring were more strongly associated with a reduction in dampening responses (i.e., downplaying positive emotions) than with increases in amplifying responses (i.e., enhancing positive affect) in a sample of older adults. There is also some evidence that savouring might only amplify affective benefits of positive events in terms of increased positive emotions when individuals experience an overall low number of positive events (Hurley and Kwon 2013; Jose et al. 2012). Thus, the role of savouring in modulating positive affect reactivity in an adaptive or maladaptive way should be further investigated among older adults.

### 7.3 Limitations and Future Directions

Although the present study utilised a daily diary methodology, which has advantages in investigating associations between daily events and well‐being (Almeida 2005), our ability to conclude about causality is limited. For example, the direction of the associations can be reversed, such that the findings related to the daily measurement of daily affect, stressors, and savouring could indicate that people are more inclined to savour if there are positive events (to be savoured) or fewer daily stressors. Nonetheless, even if savouring is affected by positive events or reduced stressors, this does not rule out the possibility that savouring could also have a subsequent effect on positive outcomes, as demonstrated in savouring‐based interventions (e.g., Ho et al. 2014; Smith and Hanni 2017). Furthermore, while experimental designs are necessary to establish whether savouring directly causes changes in affect reactivity following daily events, the consistency in the findings regarding the moderating effects of daily and trait savouring on reactivity to positive and negative events represents an important preliminary step towards this understanding. Second, participants in the present study were older adults who reside in the community, are retired, and generally maintain good health. Given that the relation between daily events and well‐being can vary depending on an individual's health status (e.g., Chiang et al. 2018; Sin et al. 2020), it is crucial to explore the research inquiries across a more diverse sample encompassing various health conditions. In addition, while savouring in the present study was measured in general terms, it is important to investigate the role of specific savouring strategies such as temporal awareness of the fleeting nature of life, counting blessings, and sharing with others (Bryant and Veroff 2017) in order to understand if and how there is a differential effect on positive and negative affect. Furthermore, future research should go beyond measures of emotional well‐being and investigate whether savouring can moderate the effect of health ramifications of daily events on health outcomes measured by stress hormones or somatic symptoms. Finally, daily reporting on savouring experiences may have increased participants' focus on positive events, possibly enhancing their savouring responses. While this could have influenced mean scores, it is unlikely to have biased our findings, as the study focused on savouring's moderating role and its associations with affective outcomes.

### 7.4 Conclusions

Overall, the findings of the present study highlight the relevance of everyday savouring and its potential to modulate affect reactivity in older adults. Specifically, daily savouring was found to be associated with both higher positive and lower negative affect. Furthermore, on a daily level, the association between negative stressors and negative affect was no longer significant on days with high levels of daily savouring, and the negative association between positive events and negative affect was only significant among individuals with high levels of trait savouring. These findings highlight the importance of distinguishing between state‐like (daily) and trait‐like savouring in understanding affective reactivity to daily events. While both forms of savouring were associated with positive affect, their moderating effects with respect to negative affect reactivity differed, reflecting distinct regulatory processes at the intraindividual and interindividual levels.

## Conflicts of Interest

The authors declare no conflicts of interest.

## Data Availability

Data is available at: https://osf.io/93kts/.

## Endnotes

^1^
In the present study, three items were used instead of four, as one item in the original scale asked whether participants had experienced any positive feelings (yes/no) and subsequently asked the three items about savouring only if participants responded ‘yes’ to the first question. In this study, we opted to avoid the use of branching and instead asked all participants about their responses to any positive experiences they had that day in all daily surveys.
